# Relationship between the Synthesis Method and the Magnetoelectric Properties of Bismuth Sodium-Potassium Titanate/Nickel Cobalt Ferrite Lead-Free Composites

**DOI:** 10.3390/ma16072759

**Published:** 2023-03-30

**Authors:** Javier Camargo, Leandro Ramajo, Miriam Castro

**Affiliations:** Institute of Research in Materials Science and Technology (INTEMA), Av. Colón 10850, Mar del Plata 7600, Argentina

**Keywords:** magnetoelectric, piezoelectric, magnetic, sol-gel, solid state, Pechini

## Abstract

In this work, the influence of the synthesis methods of piezoelectric and magnetostrictive phases on the final properties of the Bi_0.5_(Na_0.8_K_0.2_)_0.5_TiO_3_-Ni_0.5_Co_0.5_Fe_2_O_4_ composites was studied. Different routes were used to individually synthesize each phase, and the composites were prepared using different fractions for each phase. Composites were sintered, and the structural, microstructural, dielectric, and magnetoelectric properties were evaluated. According to the selected synthesis method employed for each phase, different particle sizes and reactivities of the individual phases were obtained. These differences determined the suitable sintering temperature for each set of composites and were responsible for the final properties. In fact, magnetoelectric properties were modulated by the combination of composition and synthesis routes.

## 1. Introduction

Magnetoelectric materials are able to modify magnetization and polarization by the application of an external electric or magnetic field. These materials are extensively studied due to their scientific and technological relevance in several applications [1,2,3]. In most single-phase multiferroics, the magnetoelectric (ME) coupling between electrical and magnetic ordering is generally weak and operates at very low temperatures, making it difficult to use in practical applications [4,5,6]. For example, some compounds based on the Bi_2_LaNb_1.5_Mn_0.5_O_9_ Aurivillius phase [7], the Ba_4_(Sm*_x_*La_1−*x*_)_2_Fe_2_Nb_8_O_30_ tungsten bronze phase [8] or the (Ca,Sr)_3_Mn_2_O_7_ Ruddlesden-Popper perovskites [9,10] show low magnetoelectric coupling properties even at low temperatures. On the other hand, although single-phase BiFeO_3_ ceramics are magnetoelectric at room temperature, it is difficult to obtain single-phase ceramics with acceptable properties. Therefore, the research focus has shifted towards the multiferroic compounds, which show a strong ME coupling at room temperature. These composites can be obtained through the direct mix of particles of both phases, the use of ceramic fibers, or the multilayer deposition [11,12,13,14].

Among the composites with magnetoelectric response, it is possible to find some ceramics that use lead-based materials in the piezoelectric phase, such as PbZr_0.52_Ti_0.48_O_3_ (PZT) or Pb(Mg_1/3_Nb_2/3_)_0.67_Ti_0.33_O_3_ (PMN-PT) and, for example, cobalt and nickel ferrites as the magnetostrictive phase [15]. However, in many countries, the use of lead-based materials is prohibited or limited due to their known toxicity. Consequently, in recent years different lead-free alternatives have been developed for both piezoelectric and magnetoelectric materials [16,17]. Lead-free ceramics, such as BaTiO_3_ (BT), Bi_0.5_Na_0.5_TiO_3_ (BNT), Sr_0.5_Ba_0.5_Nb_2_O_6_ (SBN), K_0.5_Na_0.5_NbO_3_ (KNN), and 82BaTiO_3_-10BaZrO_3_-8CaTiO_3_ (BZT-BCT), have been proposed for the piezoelectric phase [18,19,20,21,22,23,24,25].

Bi_0.5_Na_0.5_TiO_3_ (BNT) is a widely studied perovskite with high Curie temperature (T_c_~320 °C) and remanent polarization (P_r_~38 μC/cm^2^). Nevertheless, its high coercive field and low piezoelectric coefficient compared to other piezoceramics hinder its application [26,27]. A possible solution to considerably enhance the piezoelectric response is the introduction of BNT solid solutions. Specifically, the Bi_0.5_Na_0.5_TiO_3_ (BNT)-Bi_0.5_K_0.5_TiO_3_ (BKT) solid solution presents a morphotropic phase boundary (MPB) for the 80BNT-20BKT composition where the maximum value of the piezoelectric coefficient (d_33_), and a diminution in the value of the coercive field are reached [28,29,30,31].

In the case of magnetic materials with AB_2_O_4_ spinel structure, cobalt ferrite is a well-known hard magnetic material with relatively high coercivity (H_c_), and magnetic saturation (M_s_), whereas nickel ferrite is a soft magnetic material with low coercivity and magnetic saturation. Therefore, the combination of these hard and soft ferrites allows their use to be extended to a wide variety of applications [32,33,34]. Indeed, Ni-Co ferrites are commonly employed in electronic devices in the area of telecommunications. For example, these ferrites are used in high-quality filters, radio frequency circuits, transformer cores, and read/write heads for high-speed digital tapes. Moreover, they can be used in the miniaturization of high-frequency application devices due to the reached electromagnetic properties [35].

Another way to improve the functional properties of these ceramics is by optimizing the size of the starting powders and the size ratio between the magnetic and piezoelectric phases. It has been reported that the size of the powders plays a very important role in obtaining a certain crystalline phase and the electrical properties of ceramic pieces when these powders are in the submicron size range [36,37]. For this reason, powder synthesis methods have been extensively studied. In general, ceramic powders are prepared using the conventional solid-state reaction [38,39]. However, to ensure the complete formation of the desired phases, by this type of reaction, a long time and a high temperature (above 1100 °C) are generally required. Consequently, the synthesized powders typically have a large particle size. In contrast, chemical routes (i.e., co-precipitation technique, sol-gel process, or hydrothermal) are effective in reducing the synthesis temperature and the size of crystals in the nanometer range [38,40,41]. Additionally, it has been determined that the direct interaction between the piezoelectric and magnetic phases, without reaching the percolation limit, is essential for obtaining improvements in the magnetoelectric coefficient.

In this work, magnetoelectric compounds corresponding to the Bi_0.5_(Na_0.8_K_0.2_)_0.5_TiO_3_ (BNKT)-Ni_0.5_Co_0.5_Fe_2_O_4_ (NCF) system were prepared. The Bi_0.5_(Na_0.8_K_0.2_)_0.5_TiO_3_ (BNKT) piezoelectric phase was synthesized by two different routes: the solid-state reaction method with a mechanochemical activation step and the sol-gel method. Nevertheless, the Ni_0.5_Co_0.5_Fe_2_O_4_ (NCF) magnetostrictive phase was prepared by Pechini’s method. The compounds were prepared using different fractions of the previously synthesized phases. The dielectric, piezoelectric, and magnetic properties, as well as the magnetoelectric (ME) coefficient, were investigated. Furthermore, the ME coupling was improved by controlling the grain size to facilitate the contact between the phases and minimize the reactivity of the phases.

## 2. Materials and Methods

Two alternative routes were followed for the synthesis of the piezoelectric phase (the solid-state reaction with a mechanochemical activation step of the reagents (SS) and the sol-gel (SG) methods), whereas the magnetostrictive phase was synthesized through Pechini’s method (PE).

### 2.1. Synthesis of BNKT through the Solid-State Method (SS)

The reagents used for the synthesis were bismuth oxide, Bi_2_O_3_ (Aldrich 99.8%; Saint Louis, MO, USA), sodium carbonate, Na_2_CO_3_ (Aldrich 99.5%; USA), potassium carbonate, K_2_CO_3_ (Aldrich 99.5%; USA), and titanium oxide, TiO_2_ (Aldrich 99.9%; USA). The reagents were weighted in the stoichiometric ratio, ground in a planetary mill (Fritsch, Pulverisette 7, 1050 rpm, Idar-Oberstein, Germany) for 6 h, and then calcined at 700 °C for 2 h.

### 2.2. Synthesis of BNKT through the Sol-Gel Method (SG)

The reagents used for the synthesis were bismuth nitrate pentahydrate, Bi(NO_3_)_3_·5H_2_O (Aldrich 98%; USA), sodium acetate tetrahydrate, C_2_H_3_NaO_2_·4H_2_O (Anedra 98%; Los Troncos del Tala, BA, Argentina), potassium acetate tetrahydrate, C_2_H_3_KO_2_·4H_2_O (Biopack 98%; Argentina), and titanium butoxide, Ti(C_4_H_9_O)_4_ (Aldrich 97%; USA). Specifically, 5 mM bismuth nitrate pentahydrate, 4 mM sodium acetate tetrahydrate, and 1 mM potassium acetate tetrahydrate were separately dissolved in 25 mL acetic acid. On the other hand, 10 mM titanium butoxide, and 20 mM acetylacetone were dissolved in 12 mL 2-methoxy ethanol. All the solutions were stirred for 30 min, mixed, and finally, 2.5 mL ammonium hydroxide was added to reach pH 3. This solution was also kept at 150 °C for 24 h to obtain a fine powder. Subsequently, a heat treatment at 700 °C for 30 min was carried out to obtain the desired BNKT phase.

### 2.3. Synthesis of NCF Phase

The reagents were nickel nitrate, Ni(NO_3_)_2_·6H_2_O hexahydrate (Baker 99.8%; Phillipsburg, NJ, USA), cobalt nitrate hexahydrate, Co(NO_3_)_3_.6H_2_O (Biopack 98%, Buenos Aires, Argentina), iron nitrate nonahydrate, Fe(NO_3_)_3_·9H_2_O (Aldrich 98%; USA), and citric acid (Cicarelli 99%; Santa Fe, Argentina). The reactants were separately dissolved in a 1:1:4:6 stoichiometric ratio in water in a 1.25 molar ratio. All the solutions were stirred for 30 min, then they were mixed, and a final pH 7 was obtained by the addition of ammonium hydroxide. The final solution was reserved at 200 °C until self-combustion to obtain a fine black powder. Afterwards, in order to find the desired phase, the black powder was thermally treated at 850 °C for 2 h.

### 2.4. Composites Preparation

BNKT and NCF powders were mixed in different weight proportions according to xBi_0.5_(Na_0.8_K_0.2_)_0.5_TiO_3_-(100-x)Ni_0.5_Co_0.5_Fe_2_O_4_, x = 0, 60, 70, 80, 90, 95, and 100 in a planetary mill for 15 min. The powders were uniaxially pressed into disks 1 cm in diameter and 0.2 mm thick. Taking into account previous studies carried out on xBNKT-(100-x)NCF composites [38], the sintering temperature of the compounds was established from an analysis of the densification degree of the samples with composition 70BNKT-30NCF. For this composition, the sintering temperature was varied between 950 °C and 1125 °C. Based on this analysis, the sintering cycle for the composites was determined. BNKT (SS)-NCF (PE) samples were sintered at 1075 °C, whereas BNKT (SG)-NCF (PE) samples were sintered at 975 °C.

### 2.5. Characterization

The obtained ceramics were evaluated using several techniques (X-ray diffraction (XRD, PANalytical, X’pert Pro, CuKα), Raman spectroscopy (Renishaw in Via microscope through the 514 nm Ar-ion laser line, Wotton-under-Edge, Glos, UK), and Field Emission Scanning Electron Microscopy (Zeiss Crossbeam 350, Jena, Germany) equipped with Energy-Dispersive Spectroscopy (EDS Oxford Ulti Max 100, High Wycombe, Bucks, UK). The apparent density of the sintered composites was obtained by means of Archimedes’ method using distilled water as the immersion medium at room temperature. The theoretical density (δ) for BNKT-NCF composites was calculated from the mixture rule, using the following equation:

δ = (W_BNKT_ + W_NCF_)/(W_BNKT_/δ_BNKT_ + W_NCF_/δ_NCF_) (1)

where W_BNKT_ and W_NCF_, are the weight percentages of the BNKT and NCF in the mixture, respectively; δ_BNKT_ and δ_NCF_ are the theoretical densities of BNKT (5.97 g/cm^3^) and NCF phases (5.334 g/cm^3^). The relative density was calculated by the quotient between the apparent density and the theoretical density.

Dielectric measurements were carried out on the sintered discs painted with silver/palladium electrodes on the plain surfaces. Real permittivity and dielectric loss tangent were determined employing an impedance analyzer (HP4284A LCR meter, Agilent Technologies, Englewood, CO, USA) in a temperature range between 20 to 500 °C while the piezoelectric coefficient d_33_ was measured using a quasi-static piezoelectric d_33_ meter (YE2730—Sinoceramics, Shanghai, China).

Magnetization was measured, at room temperature and as a function of the magnetic field between +13 and −13 kOe, using a vibrating sample magnetometer (Lakeshore 7300, Westerville, OH, USA).

The magnetoelectric voltage coefficient (α_33_) of the composites was obtained from the magnetic field-induced voltage measured across the sample using a lock-in amplifier (model NF LI5640) and employing a bias magnetic field up to 15 kOe with an ac magnetic field of 2 Oe at 1 kHz.

## 3. Results

Based on a previous work carried out on BNT(SS)-NCF(SS) composites [38], the density of the 70BNT(SS)-30NCF (70-30) sample was taken as a parameter to determine the sintering temperature of each set of samples. Table 1 shows the density values of the sintered samples at different temperatures. For the SS-PE samples the maximum density value was observed when samples were sintered at 1075 °C, whereas for the SG-PE samples the maximum density value was obtained in the samples sintered at 975 °C. Consequently, all sets of samples were sintered at the temperature where the maximum density was registered. Thus, the complete SS-PE set was sintered at 1075 °C for 2 h, whereas the SG-PE set was sintered at 975 °C for 2 h.

**Figure 1:**
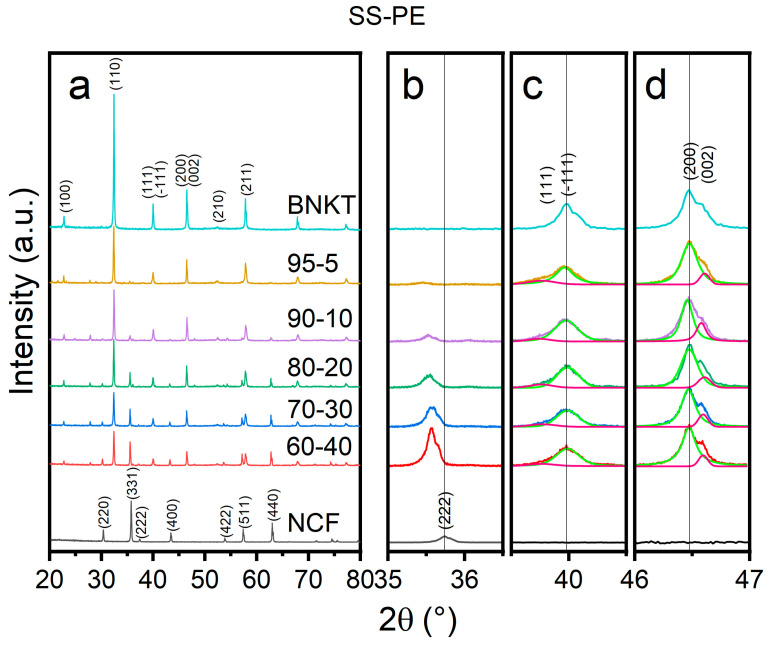
XRD patterns of the xBNKT-(100-x)NCF (x = 100, 95, 90, 80, 70, 60, 0) SS-PE composites (**a**). Enlargements of 35–36.5° (**b**), 39–41° (**c**), and 46–47° (**d**) angles.

**Figure 2:**
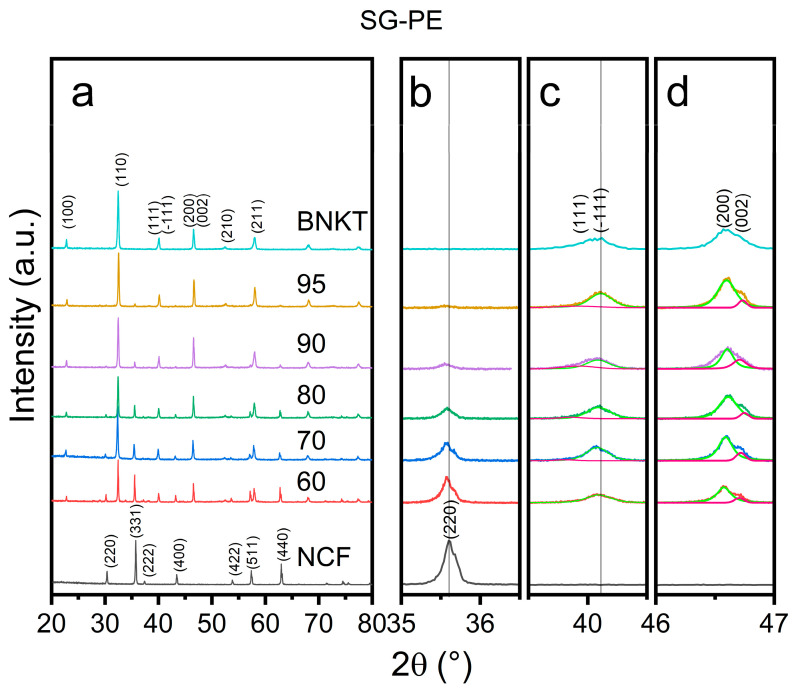
XRD patterns of the xBNKT-(100-x)NCF (x = 100, 95, 90, 80, 70, 60, 0) SG-PE composites (**a**) Enlargements of 35–36.5° (**b**), 39–41° (**c**), and 46–47° (**d**) angles.

In Figure 1 and Figure 2 the XRD patterns of the sintered samples are shown. All patterns of the composite samples exhibit the peaks assigned to the BNKT [42] and NCF (JCPDS card 04-002-0422) phases without the presence of secondary phases. Analyzing in detail the main peak of NCF (located at ~35.5°), a displacement to lower angles as the amount of BNKT increases in SS-PE samples is registered. This change can be attributed to the expansion of lattice parameters due to the diffusion of larger radius ions into smaller ion-site in the NCF phase. However, in the SG-PE samples (Figure 2), as they were sintered at a lower temperature, the ferrite remains stable, and the peak displacement is less perceptible.

For the BNKT phase, structural phase evolution can be examined by the peak splitting phenomenon of the (111) and (200) peaks. In these composite samples, the rhombohedral stabilization can be confirmed by the presence of the (−111) and (111) peaks near 41° and the combination of the (002)/(200) peaks into only one peak at 46.5°, indicating the tetragonal phase absence, for both methods. It must be pointed out that the small peak located at 46.7° belongs to K_α2_ [43].

**Figure 3:**
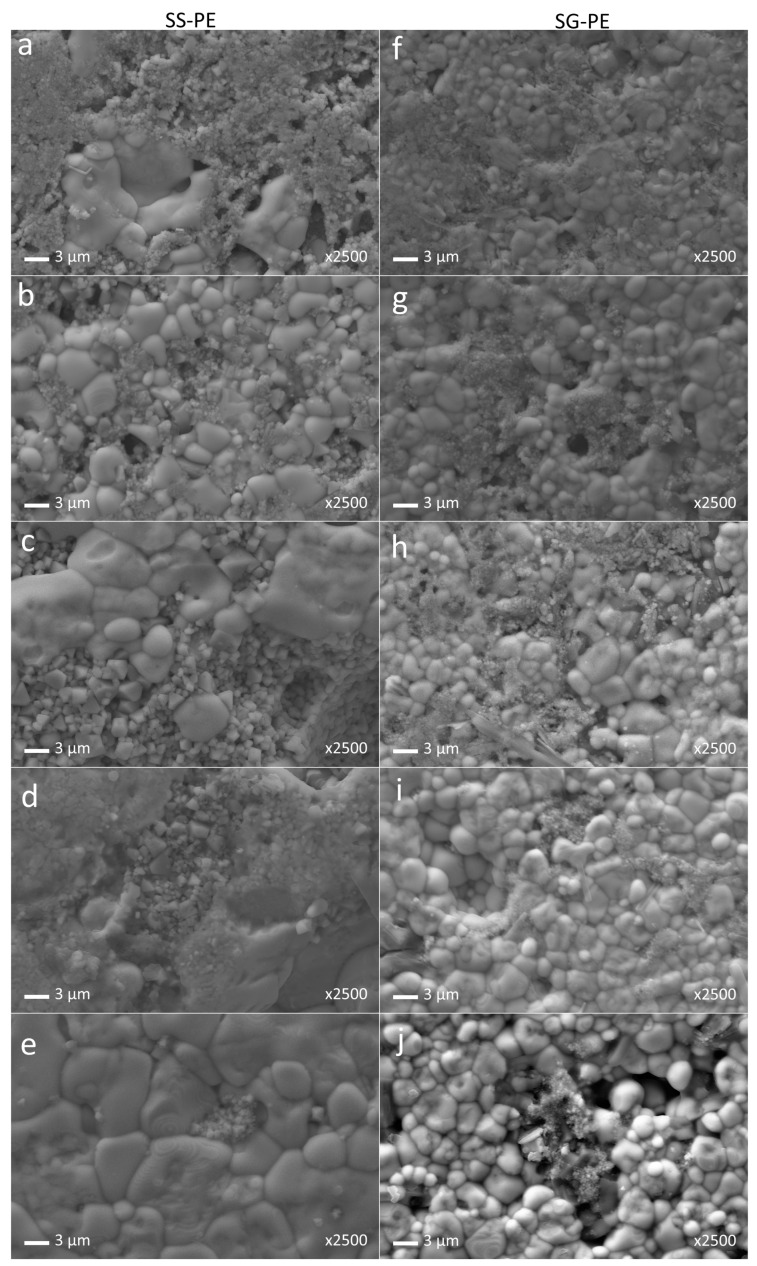
SEM images of the xBNKT-(100-x)NCF (x = 60 (**a**,**f**), 70 (**b**,**g**), 80 (**c**,**h**), 90 (**d**,**i**), 95 (**e**,**j**)) sintered composites.

Figure 3 shows the SEM images of the sintered composites. Firstly, taking into account the grain size of pure BNKT ceramics, synthesized by the solid-sate (0.7 ± 0.2 µm) or the sol-gel (0.22 ± 0.10 µm) methods [44,45], and the grain size of the BNKT phase in the 95-5 composites (BNKT SS-PE 7 ± 4 µm and BNKT SG-PE 2.4 ± 1.4 µm), considerable grain growth is registered in these composites. Prasertpalichat et al. studied the influence of Fe addition on the final properties of 0.93Bi_0.5_Na_0.5_TiO_3_-0.07BaTiO_3_ and they attributed the grain size increasing to the oxygen vacancies generated from doping [46]. Oxygen vacancies facilitate mass transportation during the sintering process and promote grain growth. Consequently, the initial grain growth of the BNKT phase by the NCF addition in the composites can be attributed to the iron diffusion into the BNKT phase. Secondly, the increasing ferrite concentration does not produce a continuous grain growth of the piezoelectric phase. Apparently, as the amount of the ferrite phase increases, the Fe diffusion into the BNKT is reduced due to the stabilization of the ferrite phase into the composites. In addition, the ferrite phase could impede the grain growth of the BNKT phase due to steric issues. Finally, the lower grain growth observed in the SG-PE than in the SS-PE set of samples can be assigned to the inferior sintering temperature required for the SG-PE set of samples and to the smaller initial particle size of the SG powder.

**Figure 4:**
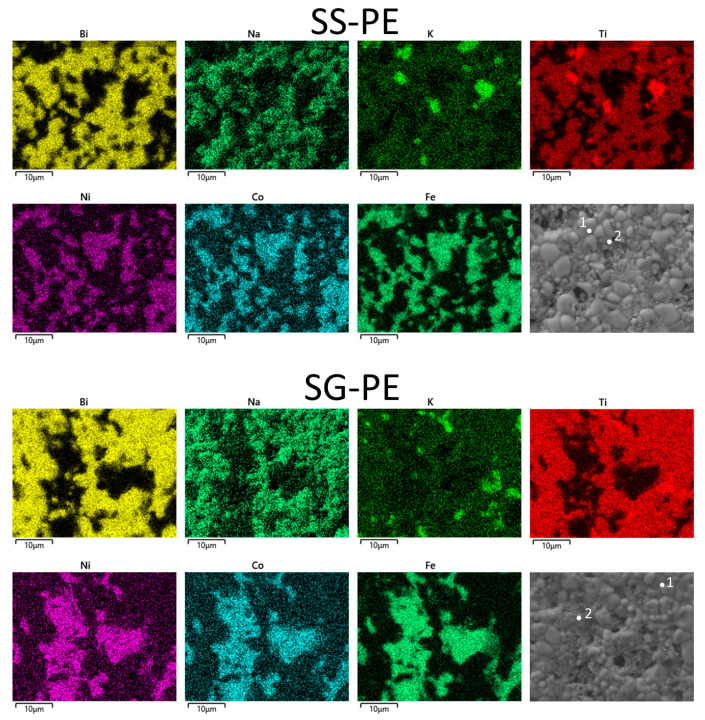
EDS mapping of the different elements corresponding to the 70BNKT-30NCF composites. Points 1 and 2 indicate the places where the atomic content was analyzed.

In Figure 4 the atomic mapping of the different elements in the 70BNKT-30NCF composites can be observed. Additionally, the atomic content corresponding to the ferrite and piezoelectric phases is reported in Table 2. Taking into account the detection limit of the technique, the final compositions obtained by both synthesis processes were in good agreement with the expected ones. Moreover, in both cases, the small iron content detected in the perovskite phase corroborates the influence of this element on the BNKT grain growth.

Table 3 shows the apparent and theoretical densities, and the densification degree values for both sets of samples. Taking into account that the sintering temperature of each set of samples was optimized for the 70-30 composition, relevant variations with composition are not observed. Although the synthesis method of the piezoelectric phase is changing (solid-state reaction or sol-gel) and therefore so is its reactivity, this effect is compensated with the different sintering temperatures (975 °C for the SG-PE and 1075 °C for the SS-PE).

**Figure 5:**
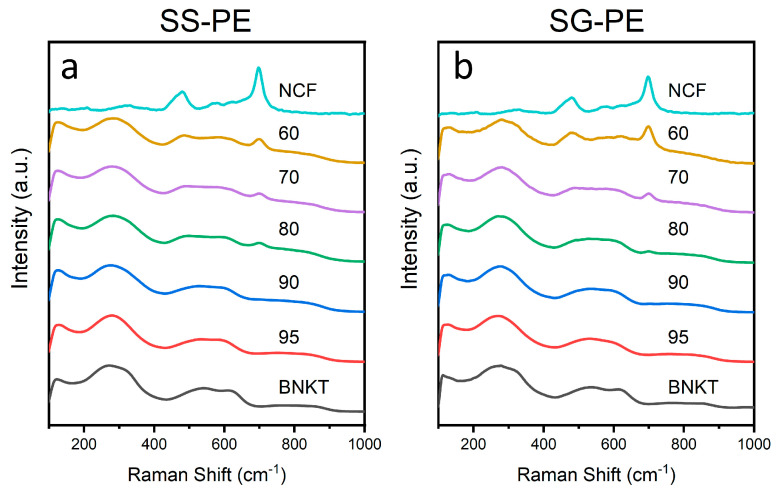
Raman spectra of xBNKT-(100-x)NCF (**a**) SS-PE and (**b**) SG-PE composites.

Figure 5 shows the Raman spectra of both sets of BNKT-NCF sintered composites, where the spectra of BNKT and NCF phases sintered at the corresponding sintering temperatures are also presented. For BNKT samples, four characteristic bands are observed. The band at 130 cm^−1^ is associated with the A-site vibration in the perovskite structure, the band around 240 and 400 cm^−1^ is associated with the Ti-O vibration, the band at 430–700 cm^−1^ is connected to the TiO_6_ octahedra vibration, and finally, the band above 700 cm^−1^ is related to A_1_ and E longitudinal optical overlapping bands [47]. For pure NCF samples, five bands in the Raman spectra are identified. The bands located at 700, 650, and 620 cm^−1^, associated with the symmetric stretch of oxygen atoms along the (Fe/M)-O bond, can be assigned to Fe-O, Ni-O, and Co-O cations, respectively. The band at 485 cm^−1^ is associated with the anti-symmetric stretch of oxygen atoms along the (Fe/M)-O bond. The bands at 327 and 570 cm^−1^ are symmetric and antisymmetric bending modes of oxygen concerning Fe(M), respectively, and finally, the band at 213 cm^−1^ is assigned to the translational movement of tetrahedron MO_4_ [48].

**Figure A1:**
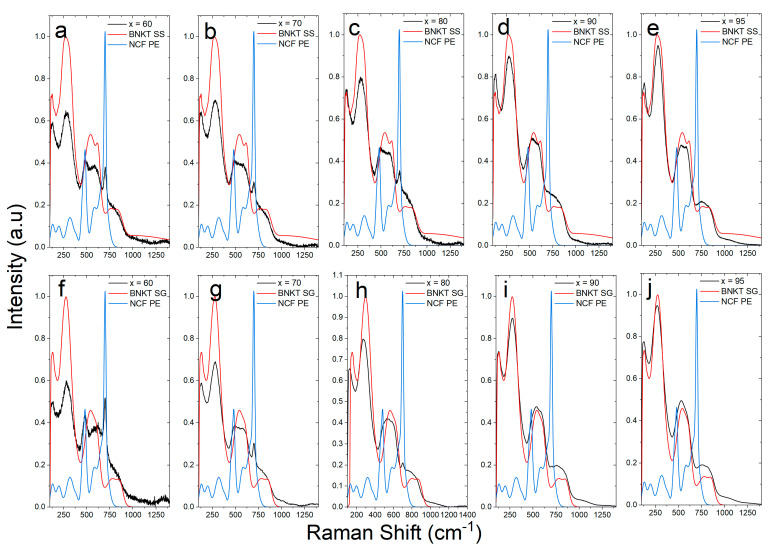
Experimental and calculated Raman spectra of xBNKT-(100-x)NCF (x = 100, 95, 90, 80, 70, 60, 0) SS-SS (**a**–**e**) and SG-PE (**f**–**j**) sintered samples.

To study in detail the influence of ferrite on the Raman spectrum of the piezoelectric phase, the spectra of the compounds were analytically calculated from the algebraic sum of the experimentally obtained spectra of the individual phases (BNKT and NCF) corresponding to the different composites. After comparing the experimental Raman spectra of the composites with the corresponding algebraic sum (see, Figure A1 in Appendix A), it is observed that the BNKT bands below 400 cm^−1^ are not affected by the ferrite addition. In the zone between 400 and 800 cm^−1^, ferrite addition modifies the TiO_6_ octahedral vibration of the BNKT phase. In addition, from the analysis of the experimental and calculated Raman spectra, it is observed that the ferrite bands are only individualized in NCF compositions of 20% or higher, in agreement with other published papers [49,50].

**Figure 6:**
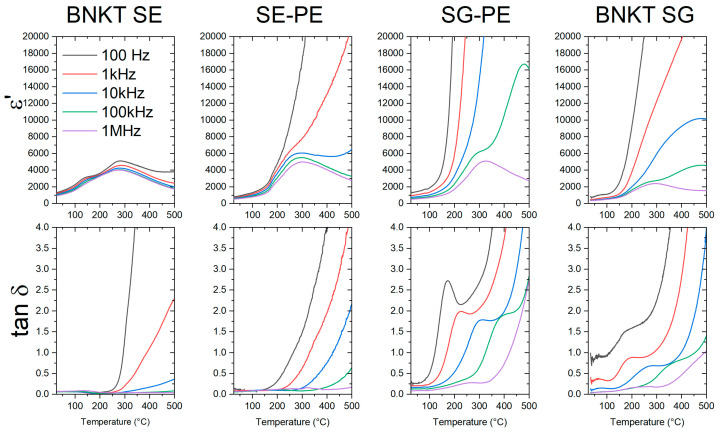
Real permittivity (ε′) and loss tangent (tanδ) vs. temperature curves at different frequencies of BNKT (SS and SG), and 95BNKT-5NCF (SS-PE and SG-PE) sintered samples.

In Figure 6 real permittivity and loss tangent vs. temperature curves at different frequencies of pure phases and composites, with 5% of NCF addition, obtained by the different methods are shown. From the figure, a strong influence of the ferrite conductivity process at higher temperatures for frequencies lower than 100 kHz is observed. This effect was also observed in the previously published results corresponding to BNKT(SS)-NCF(SS) samples [51]. These curves show two dielectric anomalies, the depolarization temperature (T_d_) existing at around 130 °C and a maximum permittivity temperature (T_m_) close to 280 °C. Moreover, the typical relaxor behavior with frequency is clearly observed in sample BNKT-SS (Figure 6). Broad peaks at T_d_ and T_m_ indicate the characteristics of a diffuse phase transition [52,53]. Interestingly, BNKT samples synthesized by the sol-gel method rendered high dielectric losses at low frequencies which could be associated with the low densification level, and the small grain size obtained after sintering at 975 °C.

**Figure 7:**
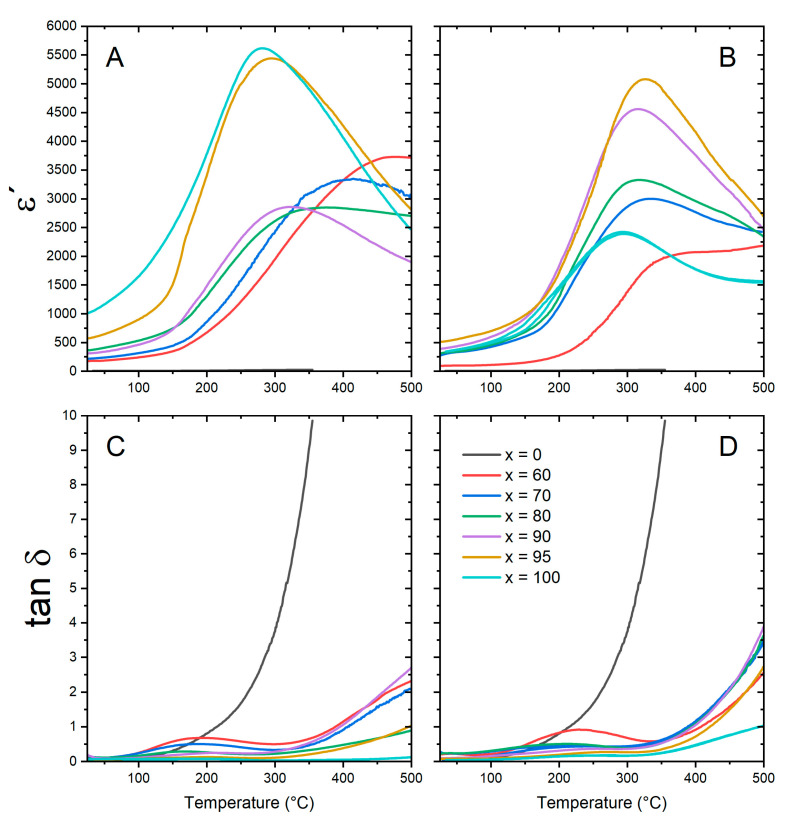
Real permittivity (ε′) and loss tangent (tanδ) vs. temperature curves for BNKT, NCF, and BNKT-NCF sintered samples obtained by the different methods at 1 MHz. SS-PE (**A**, **C**) and SG-PE (**B**, **D**).

To avoid the frequency dependence and to be able to compare the influence of the ferrite addition on dielectric properties, Figure 7 only shows the dielectric properties of both sets of samples at 1 MHz. Considering that the dielectric permittivity of the ferrite phase is lower than that of the piezoelectric phase, then the dielectric permittivity decreases when the ferrite addition increases. However, in SG-PE samples, the notable increase in the real permittivity with the addition of NCF to the pure BNKT phase can be associated with the observed grain size increment. Indeed, the low dielectric properties of the BNKT SG sample can be related to the low sintering temperature and, consequently, the small grain growth reached. In addition, the temperature corresponding to the maximum dielectric permittivity moves to higher values when the NCF content is increased, particularly for the SS-PE set of samples. On the contrary, P Gupta et al. observed a reduction in the *T_m_* with Fe addition to BaTiO_3_ and they attributed the observed behavior to the substitution of the lower valence Fe^2+^/Fe^3+^ at the Ti^4+^ site creating oxygen vacancies [46]. In these sets of samples, two possible effects influence the dielectric properties: the possible ions diffusion between both phases and the variation in the grain growth of the BNKT phase as the NCF content is increased. Additionally, for the highest temperatures, the dielectric loss of all composites increases due to the ferrite conductivity.

**Figure 8:**
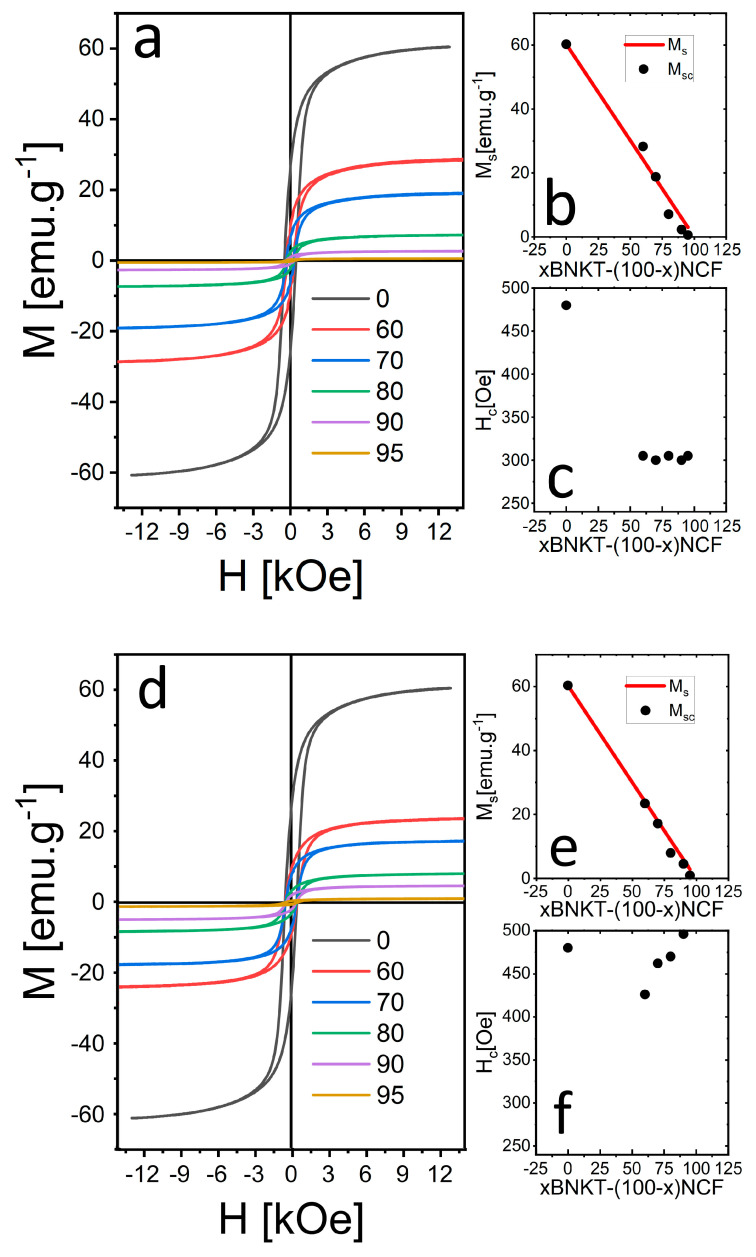
Magnetic hysteresis loops of the pure NCF sample and xBNKT-(100-x)NCF composites (**a**,**d**), Saturation magnetization (**b**,**e**) and coercive magnetic field (**c**,**f**) progress with composition.

In Figure 8, the magnetic hysteresis loops and the saturation magnetization and coercive field evolution with composition are presented. In both systems, as the BNKT phase amount increases, the saturation magnetization decreases according to Vegard’s Law. Nevertheless, the BNKT phase increment produces a different effect in the coercive magnetic field depending on the selected synthesis and processing conditions (see Table 4). These variations are associated with compositional changes and the final grain size of the magnetic phase.

**Figure 9:**
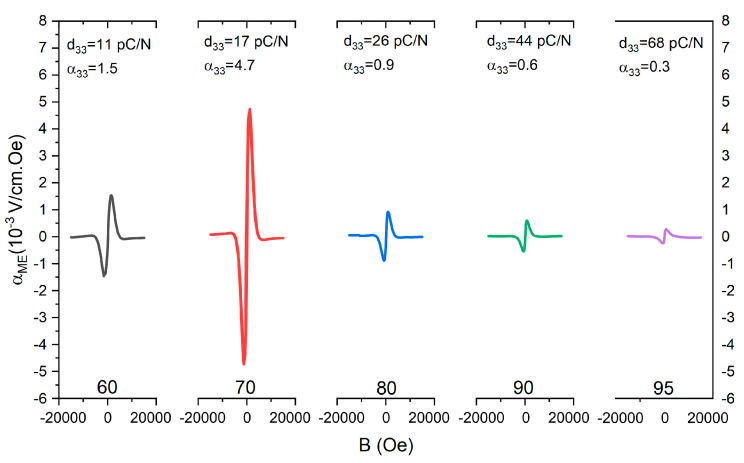
Magnetoelectric voltage coefficient (α_33_) vs. dc magnetic field (B) of SS-PE sintered composites employing an ac magnetic field of 2 Oe at 1 kHz. The piezoelectric coefficient (d_33_) of the composites is also reported in the figure.

Figure 9 shows the magnetoelectric voltage coefficient as a function of the magnetic field for the different SS-PE composites. Unfortunately, SG-PE composites were not completely polarized as a consequence of the small grain size of the magnetic phase due to the low sintering temperature required to avoid the possible reaction or the loss of the BNKT volatile elements. Consequently, the magnetoelectric voltage coefficient could not be obtained for this set of samples. From the figure, the highest magnetoelectric voltage coefficient is registered for the 70BNKT-30NCF composite. For higher NCF concentrations, the magnetoelectric voltage coefficient decreases. In composites, the ME voltage coefficient is determined by the conjunction between piezoelectric, magnetostrictive, and microstructural properties [51]. In addition, it can be seen that the highest value of the magnetoelectric coefficient is obtained for the composition with a 30% ferrite phase. Interestingly, comparing the magnetoelectric voltage coefficient values obtained in the SS-PE composites with those previously reported for compounds of the same composition but with both phases obtained by the solid-state reaction (SS) method and sintered at 1100 °C, it is observed that the maximum magnetoelectric voltage coefficient is also found at 30% of magnetic phase and the observed values are similar to those found here (Table 5). Considering that in SS-SS and SS-PE, the selected sintering temperature is not enough for the NCF grain growth, the characteristics of the BNKT phase and the overall microstructure determine the magnetoelectric properties. Additionally, as was expected, the higher the amount of the NCF phase, the smaller the value of the piezoelectric coefficient. From these results, it is possible to affirm that the selected synthesis method used for the piezoelectric or ferrite phases determines the final properties of the composites. In fact, magnetoelectric properties are strongly dependent on the possible diffusion or reaction between both phases. Specifically, comparing composites of the same composition but with both phases obtained from the solid-state reaction method Table 5, the use of ferrite powder obtained by Pechini’s method allowed for reducing the calcination and sintering temperatures for NCF obtained from the solid-state reaction method T_cal_ = 1050 °C and for SS-SS composites T_s_ = 1100 °C during 5 h [51], whereas for NCF obtained from Pechini’s method T_cal_ = 700 °C and for SS-PE composites T_s_ = 1075 °C during 2 h, maintaining the magnetoelectric properties. Moreover, an improvement in the magnetoelectric properties for x = 60 composites is registered for SS-PE samples. This variation could be associated with a better distribution of the phases. Finally, it is necessary to pointed out that the magnetoelectric coupling coefficients here presented are in good agreement with those found for other lead-free piezoelectric-ferrite particulate composites (2.02 mVcm^−1^Oe^−1^ for 20 K_0.5_Na_0.5_NbO_3_/80 MnFe_2_O_4_ [54], and 4.875 mVcm^−1^Oe^−1^ for 60Na_0.5_Bi_0.5_TiO_3_-40CoCr_0.4_Fe_1.6_O_4_ composite [55]), where for better magnetoelectric coupling coefficient values laminates composites [56] or films [57] are required.

## 4. Conclusions

Bi_0.5_(Na_0.8_K_0.2_)_0.5_TiO_3_ (BNKT) powders were successfully synthesized by two alternative methods (solid-state reaction (SS) and sol-gel (SG)), whereas Ni_0.5_Co_0.5_Fe_2_O_4_ (NCF) powders were obtained by the Pechini’s method (PE). Composites containing different amounts of the ferrite phase, and free of secondary phases, were obtained for both sets of samples (SS-PE and SG-PE). Trying to avoid the possible reaction between both phases and the decomposition of the piezoelectric phase, the sintering temperature of each set of samples was selected according to the final density of the sintered 70-30 composites. Consequently, in order to obtain similar final densities, SG-PE set samples were sintered at 975 °C, while samples of the SS-PE set were sintered at 1075 °C. By XRD and Raman spectroscopy, only the piezoelectric and ferrite phases were confirmed. Nevertheless, in the samples sintered at the highest temperature (SS-PE set) changes in the position of the XRD peaks can be assigned to variations in the lattice parameters of the ferrite phase due to the diffusion of the ions between both phases. In both sets of samples, an increase in the average grain size was registered for the lowest ferrite amount due to the possible iron diffusion in the piezoelectric phase. However, for the other compositions, this rise in the BNKT grain size was not increased. Although magnetization values of both sets of samples follow Vegard’s Law, the magnetoelectric coefficient was strongly dependent on the selected synthesized method. SS-PE composites rendered similar magnetoelectric properties than composites, previously reported, made off both powders obtained by the solid-state reaction method, whereas for SG-PE composites the polarization of the samples was reduced due to the highest samples conductivity. In summary, to improve magnetoelectric properties, the possible diffusion or reaction between both phases must be avoided. Consequently, the selected synthesis method for the obtention of the piezoelectric or ferrite phases determines the final properties of the composites. In this case, when comparing with composites of the same composition but with both phases obtained from the solid-state reaction method, the use of ferrite powder obtained by Pechini’s method was a valuable alternative to reduce the calcination and sintering temperatures, without reducing the magnetoelectric properties.

## Figures and Tables

**Table 1:** Experimental and relative density values of 70BNKT-30NCF (SS-PE and SG-PE) samples sintered at different temperatures.

70BNKT-30NCF SS-PE Samples			70BNKT-30NCF SG-PE Samples		
Temperature (°C)	Density (g/cm^3^)	Relative Density (%)	Temperature (°C)	Density (g/cm^3^)	Relative Density (%)
975	5.15 ± 0.10	89.3	950	5.16 ± 0.02	89.5
1000	5.13 ± 0.07	89.0	975	5.23 ± 0.04	90.7
1025	5.13 ± 0.08	89.0	1000	5.04 ± 0.08	87.4
1050	5.17 ± 0.08	89.6	1025	4.98 ± 0.08	86.3
1075	5.18 ± 0.07	89.8	1050	4.60 ± 0.20	80.4
1100	4.40 ± 0.20	76.8	1075	4.39 ± 0.01	76.2
1125	4.20 ± 0.10	73.1	1100	4.10 ± 0.10	70.4

**Table 2:** Atomic content determined by EDS of the 70BNKT-30NCF SS-PE and SG-PE composites.

**70BNKT-30NCF SS-PE**														
**Point 1**							**Point 2**							
Bi	Na	K	Ti	O	Fe		Ni	Co	Fe	O	Bi	Na	K	Ti
0.23	0.16	0.02	0.42	1.54	0.04		0.21	0.21	0.74	1.72	0.01	0.00	0.00	0.06
**70BNKT-30NCF SG-PE**														
**Point 1**							**Point 2**							
Bi	Na	K	Ti	O	Fe		Ni	Co	Fe	O	Bi	Na	K	Ti
0.23	0.15	0.02	0.43	1.55	0.03		0.22	0.22	0.73	1.77	0.01	0.02	0.00	0.07

**Table 3:** Apparent, and theoretical densities, and densification degree values of BNKT-NCF (SS-PE and SG-PE) samples.

xBNKT-(100-x)NCF (x)	Theoretical Density (g/cm^3^)	SS-PE (Sintered a 1075 °C)		SG-PE (Sintered at 975 °C)	
		Density (g/cm^3^)	Densification Degree (%)	Density (g/cm^3^)	Densification Degree (%)
60	5.70	5.14 ± 0.04	90.2	5.07 ± 0.07	89.0
70	5.76	5.18 ± 0.07	89.8	5.23 ± 0.04	90.7
80	5.83	5.27 ± 0.06	90.4	5.26 ± 0.04	90.8
90	5.90	5.27 ± 0.10	89.3	5.34 ± 0.09	90.5
95	5.93	5.51 ± 0.02	92.9	5.33 ± 0.08	89.8

**Table 4:** Experimental (M_s_) and calculated (M_sc_) saturation magnetization and coercive field (H_c_) of xBNKT-(100-x)NCF (x = 95, 90, 80, 70, 60, 0) SS-PE and SG-PE composites.

Sample	SS-PE Samples			SG-PE Samples		
	M_s_ (emu/g)	M_sc_ (emu/g)	H_c_ (Oe)	M_s_ (emu/g)	M_sc_ (emu/g)	H_c_ (Oe)
0	60.3	-	480	60.3	-	480
60	28.3	24.2	260	23.5	24.1	426
70	18.8	18.1	300	17.3	18.1	462
80	7.1	12.1	305	8.1	12.1	470
90	2.3	6.0	300	4.7	6.0	496
95	0.6	3.0	305	1.1	3.0	512

**Table 5:** Magnetoelectric voltage coefficient (α_33_) and piezoelectric coefficient (d_33_) of SS-SS [51] and SS-PE composites.

Sample	α_33_ (mV/cmOe)		d_33_ (pC/N)	
	SS-SS	SS-PE	SS-SS	SS-PE
60	0.69	1.5	10	11
70	4.81	4.7	24	17
80	2.3	0.9	28	26

## Data Availability

Not applicable.
